# Clinical trials of antibody drugs in the treatments of atopic dermatitis

**DOI:** 10.3389/fmed.2023.1229539

**Published:** 2023-09-04

**Authors:** Guihao Zhou, Yueyao Huang, Ming Chu

**Affiliations:** Department of Immunology, School of Basic Medical Sciences, National Health Commission (NHC) Key Laboratory of Medical Immunology, Peking University, Beijing, China

**Keywords:** atopic dermatitis, antibody drugs, clinical trials, dupilumab, tralokinumab

## Abstract

Atopic dermatitis (AD) is one of the most common, relapsing, chronic inflammatory skin disease, being regarded as a global health issue. Recent studies have shown that Th2 cell-mediated type 2 immunity plays a central role in AD. The type 2 inflammatory cytokines such as IL-4, IL-13, IL-22, IL-31, IL-17 and IL-5 mediate the pathogenesis of AD. A variety of antibody drugs targeting these cytokines have been developed to treat AD in clinics. Notably, several antibody drugs have exhibited high efficacy in treating atopic dermatitis in previous studies, demonstrating that they could be therapeutic methods for AD patients. Herein, we reviewed the clinical trials of antibody drugs in the treatment of AD, which provides a useful guideline for clinicians to treat patients with AD in clinics.

## Introduction

Atopic dermatitis (AD), also known as atopic eczema, is a chronic relapsing inflammatory skin disorder (1, 2). Clinical symptoms of atopic dermatitis are obviously dry skin, erythema, itching, and intense pruritus (3). It is reported that AD affects approximately 25% world population in various ages, involving 10–20% of children and 10% of adults (4). Moreover, AD is able to develop into other atopic diseases such as food allergy, allergic rhinitis and asthma, affecting the quality of work and life (5). Emerging evidence revealed that AD is a type 2 inflammation mainly mediated by Th2 cells (6–8). During the allergic response, dendritic cells and macrophages can activate Th2 cells by production of thymic stromal lymphopoietin (TSLP). Then, Th2 cells will secret a large amount of type 2 cytokines such as IL-4, IL-5, IL-13, IL-22 and IL-31, leading to a type 2 inflammation (9).

Based on the mechanism of atopic dermatitis, various treatments have been developed for patients at different stages (10). Current treatments could provide symptomatic and temporary relief, however, they have limited effects for moderate-to-severe AD patient (11). Thus, antibody drugs have been researched and developed in these decades (12). These drugs target specific cytokines, including IL-4/IL-13, IL-22 and Immunoglobin E (IgE). In addition, other monoclonal antibody targeting IL-17, IL-23, OX40 and TSLP are currently under investigation, while a number of them have already entered clinical trials (13). Herein, we review the research progresses in the clinical trials of antibody drugs against atopic dermatitis.

## Anti-IL- 4/13 antibody drugs

**Figure 1:**
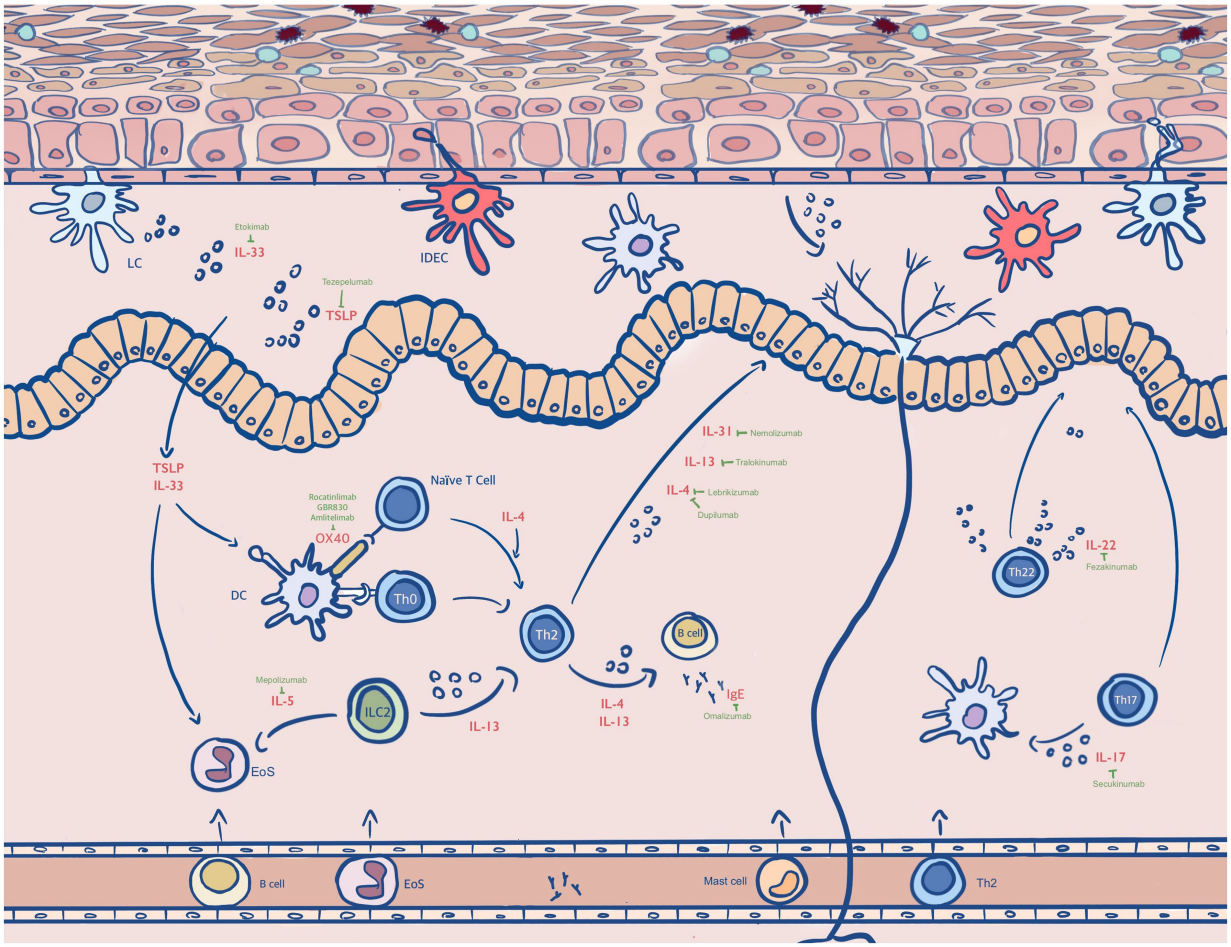
Antibody drugs in the treatments of AD. Type 2 immunity plays a central role in AD. The dendritic cell (DC), inflammatory dendritic epidermal cell (IDEC), eosinophil (EoS) and dermal dendritic cell (dDC), naïve T cell, T helper (Th) lymphocytes involve in type 2 inflammatory disorders (16). The cytokines that are secreted by these cells including IL-4, IL-13, IL-31, IL-33, IL-17, IL-22 and IL-5 mediate the pathogenesis of AD. Corresponding drugs that inhibit the effects of cytokines are Dupilumab, Tralokinumab, Lebrikilumab, Fezakinumab, Omakizumab, Nemokizumab and Fezepelumab. Others shown in this diagram are OX40, IgE, thymic stromal lymphopoietin (TSLP) and antigens, which are found to be highly expressed in atopic dermatitis.

IL-4 /IL-13 inhibitors have been identified as a milestone in treating patients with moderate-to-severe atopic dermatitis, as the type 2 inflammation, which is mediated by IL-4 and IL-13, plays a crucial role in AD (14). Dupilumab is a monoclonal antibody that block IL-4 and IL-13 signaling pathways through IL-4 receptor alpha subunit inhibition, which is the first human monoclonal antibody that has already approved by FDA for the treatment of moderate to severe atopic dermatitis in adult patients [(15); Figure 1]. This drug showed high efficacy in double-blind, randomized, placebo-controlled phase III studies (17–19), which is reflected in the improvement of Eczema Area and Severity Index (EASI) score, SCORing Atopic Dermatitis (SCORAD), and Peak Pruritus Numeric Rating Scale (PP-NRS) score (20–25). In 2014, Beck et al. found that 85% of patients in the dupilumab group (26), as compared with 35% of those in the placebo group, had a 50% reduction in the EASI score (see Supplementary Table S1). Meanwhile, more patients in the drug-treated group had a score of 0 to 1 on the IGA, comparing with those in placebo group [40% vs. 7% (*p* < 0.001)]; and pruritus scores decreased by 55.7% in the dupilumab group versus 15.1% in the placebo group (*p* < 0.001). In addition, Zhao et al. evaluated the efficacy and safety of Dupilumab in 165 adult Chinese patients with moderate-to-sever AD in a phase III study (25), and found that compared with placebo, higher proportions of patients in the dupilumab group achieved ≥75% reduction in the EASI score (57·3% vs. 14·5%) (Table 1). Meanwhile, the reductions in weekly average daily peak daily pruritus NRS were ≥ 3-point (52·4% vs. 9·6%) and ≥ 4-point (39·0% vs. 4·8%, all *p* < 0.001) respectively (Supplementary Table S1). Another phase 3 trial, which was conducted by Paller et al., investigated the efficacy of Dupilumab on children who were aged 6 months or younger (27). The given result revealed that there was considerably higher percentage of drug-treated patients had IGA 0/1 than those with placebo (28% vs. 4%), and the trend for EASI-75 was the same (53% vs.11%, *p* < 0.0001) (Table 1; Supplementary Table S1). Noticeably, numerous data showed that dupilumab still has adverse events (45) Ocular surface diseases, such as conjunctivitis (46), are typical adverse events, which was reported higher in Dupilumab group than the placebo group (5% vs. 0%) in the trial by Paller et al. (27). Others include nasopharyngitis, upper respiratory tract infection, oral herpes, injection-site reaction, and headache (17). Moreover, transient eosinophilia was also reported, which was evidenced by a significant increase in absolute eosinophil count (47). Surprisingly, while improvements of pre-existing facial dermatitis, which was suggested to be accociated with the evaluation of Malassezia-specific IgE (48), was seen after Dupilumab therapy, 24% patients reported new-onset facial erythema after the treatment (49). Similar pattern could be observed in the trial conducted by Jo et al., with totally 8 worsening cases within 101 patients, and another study carried out by de et al., which demonstrated that erythema, particularly in head and neck areas, was highly expressed after treated by Dupilumab (50, 51). Thus, despite of the relatively high efficacy of Dupilumab, it is essential to develop new drugs with milder adverse events.

**Table 1:** Efficacy of antibody drugs for treatment of atopic dermatits (AD) in clinical trials.

Target	Antibody drugs	EASI-50	EASI-75	IGA 0/1	Adverse events	Reference
IL-4/IL-13	Dupilumab	85% vs. 35% 70.7% vs. 28.9% 69% vs. 20%	62% vs. 15% 57.3% vs. 14.5% 53% vs. 11%	40% vs. 7% 26.8% vs. 4.8% 28% vs. 4%	Conjunctivitis, nasopharyngitis, upper respiratory tract infection, oral herpes, injection-site reaction, headache, transient eosinophilia	(26) (25) (27)
IL-13	Tralokinumab	73.4% vs. 51.9% N.A.	42.5% vs. 15.5% 33.2% vs. 11.4%	N.A. 22.2% vs. 10.9%	Conjunctivitis, keratitis, headache, upper respiratory tract infection, inject-site reation, keratoconjunctivitis	(28) (29)
	Lebrikizumab	82.4% vs. 62.3% 81.0% vs. 45.8%	54.9% vs. 34.0% 60.6% vs. 24.3%	33.3% vs. 18.9% 44.6% vs. 15.3%	Conjunctivitis, injection-site reaction, headache, nasopharyngitis, upper respiratory tract infection, herpesvirus infection	(30) (31)
	Eblasakimab	85% vs. 40%	67.0% vs. 0%	22.0% vs. 0%	N.A.	
IL-22	Fezakinumab	N.A.	N.A.	15% vs. 5%	Upper respiratory tract infection	(32)
IL-31	Nemolizumab	53.7% vs. 39.7% N.A. 79.7% vs. 75.0%	43.6% vs. 20.9% 50.0% vs. 15.9% 66.4% vs. 59.7%	N.A. 32.0% vs. 6.8% 28.7% vs. 16.7%	Injection-site reaction, upper respiratory tract infection	(33) (34) (35)
IL-33	Etokimab	83.3%	33%	25%	Headache, upper respiratory tract infection, conjunctivitis	(36)
IGE	Omalizumab	N.A.	N.A.	N.A.	Infective episodes, AD exacerbation, respiratory events	(37)
TSLP	Tezepelumab	64.7% vs. 48.2%	36.9% vs. 21.9%	29.4% vs. 12.9%	Nasopharyngitis, injection-site erythema, diarrhea, upper respiratory tract infection, headache	(38)
IL-17	Secukinumab	N.A.	N.A.	N.A.	orbital cellulitis, upper respiratory infection, streptococcal pharyngitis	(39)
IL-5	Mepolizumab (week 12)	45% vs. 18%	36% vs. 9%	11% vs. 0%	N.A.	(40)
OX40	Rocatinlimab	65%	55%	35%	Pyrexia, chills, aphthous ulcer, nasopharyngitis, erythema, hordeolum	(41)
		69% vs. 30%	54% vs. 11%	31% vs. 2%		(42)
	GBR830	76.9% vs. 37.5%	42.3% vs. 25.0%	23.1% vs. 12.5%	Headache, nasopharyngitis, upper respiratory tract infection, postprocedual infection, fatigue, myalgia	(43)
	Amlitelimab	N.A.	59% vs. 25%	N.A.	Infected dermal cyst	(44)

Tralokinumab is an antibody drug that specifically binds with high affinity to IL-13, inhibiting its interaction with the IL-13 receptor and thereby neutralizing the biological activity of the cytokine [(52); Figure 1]. Interestingly, the incidence of eye adverse events (conjunctivitis, keratoconjunctivitis, keratitis) caused by tralokinumab, however, was higher than that caused by Dupilumab (53). Wollenberg et al. did a phase 2b, randomized, double-blind, placebo-controlled, dose-ranging study with 204 adults moderate-to-severe AD patients to evaluate the effects of tralokinumab (28). As shown in Table 1, there is a greater percentage of tralokinumab-treated participants achieved a reduction of 50% in EASI (73.4% vs. 51.9%), and a reduction of 75% (42.5% vs. 15.5%). Meanwhile, a 50% decrease in SCORAD score could be seen more in tralokinumab group than placebo group, which was 44.2% vs. 19.5% (Supplementary Table S1 in supplementary appendix). In another randomized, double-blind, multicentre, placebo-controlled phase III trial by A Wollenberg et al., more patients who received tralokinumab achieved an IGA score of 0 or 1 than placebo in ECZTRA 1 (15.8% vs. 7.1%; *p* = 0·002) and 22·2% vs. 10·9% in ECZTRA 2 (29). While the proportion of patients achieving EASI 75 was 25.0% vs. 12.7% and 33.2% vs. 11.4% respectively, the changes in SCORAD were − 25.2 vs. -14.7 in ECATRA 1 and − 28.1 vs. -14.0 in ECZTRA 2. Improvements in pruritus, sleep interference and DLQI were observed, and these responses maintained at week 52. Noticeably, tralokinumab has recently been approved by EU for the treatment of moderate-to-severe atopic dermatitis in adult patients who are candidates for systemic therapy (54). With regard to the side effects, 17.6% of the participants experienced headache and upper respiratory tract infection, while reactions at the injection site were suffered by 5.2% of the group injected with Tralokinumab. Conjunctivitis was also experienced by 2% of the Tralokinumab group who had a 45 mg dose and 5.9% of those who had the 150 mg dose (28).

In addition, Lebrikizumab is another humanized mAb against IL-13 which has passed phase 2 trials (55). Comparing with the other monoclonal antibodies that targeted IL-13, the probability of adverse effects caused by this drug is lower (56). For instance, there is a potentially lower rate of conjunctivitis (1.4–3.8%) in lebrikinumab-treated patients compared to those used dupilumab. A randomized, placebo-controlled, double-blind phase II trial conducted by Simpson et al. indicated that among 209 adults with moderate-to-severe AD, a significant higher percentage of patients in drug-treated group achieved EASI-50 than those in placebo group (30). As demonstrated in Supplementary Table S1, other scores also show a similar trend. Later in 2020, Guttman-Yassky et al. carried out a phase 2b, double-blind, placebo-controlled, dose-ranging randomized clinical trial (31). Patients were divided into groups with different dose (placebo, 125 mg Q4W, 250 mg Q4W, 250 mg Q2W) in this study. All groups achieved a significant improvement in EASI in primary end point, with lebrikizumab group showing the most considerable reduction. As for the secondary end point, patients treating with drug had a higher percentage of improvement in IGA 0/1, EASI50, EASI75, EASI90 and NRS compared with placebo group (see Supplementary Table S1). Common TEAEs included conjunctivitis, upper respiratory tract infection, nasopharyngitis, headache, injection site reaction and injection site pain (57).

Currently, a new antibody drug targeting IL-13Ra1, Eblasakimab, was shown to be well tolerated and indicated a great improvement in a phase 1 study in 2021. At the end of this study, 89% of patients in Eblasakimab group and 40% of those in placebo group achieved EASI-50, whereas 67% achieved EASI-75 versus 0% on placebo, and the percentage of patients achieved EASI-90 was 56% vs. 0% (Table 1; Supplementary Table S1). In addition, more patients treated with Eblasakimab achieved IGA of 0/1 than patients on placebo (22% vs. 0%).

## Anti-IL-22 antibody drugs

The elevation of IL-22 concentration is correlated with the proliferation of keratinocytes and epidermal hyperplasia, hence contributing to the pathogenesis of atopic dermatits (58). Fezakinumab is a fully human monoclonal antibody against IL-22. At present, relevant clinical trials have been conducted to Phase IIa. In 2018, a randomized, double-blind, placebo-controlled trial was performed by Emma et al., which confirmed that Fezakinumab was well-tolerated, with sustained clinical improvements after drug dosing (32). The results from this study showed a significantly reduction in SCORAD in the drug-treated patients than placebo-treated patients at 12 weeks (21.6 ± 3.8 vs. 9.6 ± 4.2, *p* = 0.029) and 20 weeks (27.4 ± 3.9 vs. 11.5 ± 5.1, *p* = 0.010). Meanwhile, the improvements in body surface area were considerably higher compared to placebo-treated patients (12.4% ± 2.4 vs. 6.2% ± 2.7; *p* = 0.009), as well as the decline in IGA versus placebo groups (0.7 ± 0.2 vs. 0.3 ± 0.1; *p* = 0.034) (Table 1). Brunner et al. in the following year evaluated the cellular and molecular effects of IL-22 blockade in tissues from 60 patients with moderate-to-severe AD, and found that fezakinumab-treated patients had greater reversal of AD genomic profile than those were given placebo (25.3% vs. 10.5% at 4 weeks [*p* = 1.7 × 10^−5^]; and 65.5% vs. 13.9% at 12 weeks [*p* = 9.5 × 10^−19^]) (59). The present shown adverse events were upper respiratory tract infections (32).

## Anti-IL-31 antibody drugs

TH2 cell-released IL-31 is a critical mediator in patients with atopic dermatitis, which maximizes itch-inducing signals and causes sustained pruritus (60, 61). Nemolizumab is a humanized monoclonal antibody, which binds to signaling receptor IL-31RA to inhibit subsequent IL-31 signaling [(62); Figure 1]. Nemolizumab demonstrates a great efficacy in reducing pruritus, while one advantage of it is its quick speed of action (63). A randomized, double-blind, placebo-controlled phase 2 trial was published by Ruzicka et al. (33). Patients were given by 0.1 mg, 0.5 mg, 2 mg per kg of body weight of nemolizumab or placebo every 4 weeks. At the end of this study, changes on pruritus visual analog scale (VAS) were more significant in nemolizumab group than placebo group. [−43.7% in the 0.1-mg group, −59.8% in the 0.5-mg group, and − 63.1% in the 2.0-mg group, versus −20.9% in the placebo group (*p* < 0.01 for all comparisons)]. Changes on the EASI were − 23.0, −42.3, and − 40.9%, respectively, in the nemolizumab groups, vs. −26.6% in the placebo group, whereas the respective changes in body-surface area affected by atopic dermatitis were − 7.5, −20.0, and − 19.4% with nemolizumab, versus −15.7% with placebo. In 2018, Nemolizumab reached to second phase, Silverberg et al., in 2021, conducted a randomized phase 2B trial in order to compare its efficacy with multiple dimensions (34). According to the results, PP-NRS ≥ 4-point response of itch was observed in 68.0% nemolizumab vs. 15.9% placebo patients (*p* ≤ 0.001) at week 16, whereas the sleep disturbance was improved significantly(−26.6% to −76.0% vs. −9% to −36.5%; *p* < 0.001). Furthermore, the least square mean EASI score at the end of the trial was reduced by 68.6% in drug-treated group as compared with 42.6% in placebo group (*p* = 0.002). Later, two long-term phase III studies carried by Kabashima et al. showed clinically meaningful improvements in nemolizumab patients (35). In this trial, patients were divided into three groups, including Study-JP01, Study-JP02 and placebo group. Patients enrolled in Study-JP01 first received 60 mg nemolizumab or placebo Q4W for 16 weeks, followed by a 52 week extension period. Others who were in Study-JP02 received 60 mg Q4W up to week 52. As be seen from Supplementary Table S1, the decrease from baseline in pruritus visual VAS was 65.9% at week 68, and a similar trend could be seen in the change of EASI score (decrease by 78.2%). In terms of the adverse events caused by nemolizumab, they were normally injection-related reactions (8% vs. 3%), upper respiratory tract infection, while worsening atopic dermatitis was reported by 24% of patient in nemolizumab group and 21% of those in placebo group (64).

## Anti-IL-33 antibody drugs

IL-33 is an inflammatory cytokine that is over-expressed in keratinocytes of AD patients, stimulating group 2 innate lymphoid cells and inducing IL-31 to promote pruritus and other AD-liked phenotypes (65). Etokimab is a humanized IgG1/kappa anti–IL-33 monoclonal antibody. Chen et al. performed a phase 2a study to evaluate its efficacy and safety (36). Fortunately, all 12 patients in this trial achieved EASI50, with a 62% improvement in mean EASI being seen after 57 days. The SCORAD also reduced significantly by 40%, whereas 25% of patients reached IGA 0/1. In this trial, AEs were mostly mild and not related to Etokimab.

## Anti-IgE antibody drugs

The elevated level of total serum IgE is one major hallmark of pruritus skin disorder, especially for AD (66, 67). Omalizumab targets the high-affinity receptor binding site on IgE, acting as a monoclonal antibody drug for AD patients (68). It has been approved by FDA for asthma since 2003, and was tested for its efficacy on atopic dermatitis in recent years (69). A randomized, double-blind, placebo-controlled trial done by Iyengar et al. investigated whether omalizumab be able to modulate the allergic responses medicated by TSLP pathway in young patients between 4 and 22 years old (37). The given result showed a significant decrease in the level of TSLP, OX40L and TARC (50–75%, 70–80% and 60–80% respectively). Nevertheless, while a 20–50% reduction in SCORAD could be seen in omalizumab group, the reduction in placebo group is 45–80% (Supplementary Table S1). In 2020, a 24 week single-center, double-blind, placebo-controlled randomized clinical trial for 62 children was reported by Chan et al. (70). Based on this study, the mean difference in objective SCORAD index improvement between groups at week 24 was −6.9, whereas the difference for EASI was −6.7. In addition, improved scores of quality-of-life were seen in drug-treated group, measuring by Children’s Dermatology Life Quality Index (−3.5; 95% CI, −6.4 to −0.5) and Pediatric Allergic Disease Quality of Life Questionnaire score (−0.5; 95% CI, −0.9 to −0.0). In this trial, the number of participants with 1 or more infective episodes of AD (20% in omalizumab group vs. 25% in the placebo group) and AD exacerbation (17% vs. 19%) was low. Serious AEs occurred slightly higher proportion in omalizumab group than placebo group (20% vs. 19%), however, fewer respiratory and dermatological events were reported in drug-treated group (70). The trial from Gevaert et al. also revealed the same trend, with a higher percentage of the patients treated with omalizumab experienced SAEs than those treated with placebo, which was 2.2% vs. 1.5% (71). Thereby, the efficacy and safety of Omalizumab still need to be investigated and confirmed in the future studies.

## Anti-TSLP antibody drugs

Thymic stromal lymphopoietin (TSLP) is highly expressed in human cutaneous epithelial cells in AD patients, however, this overexpression triggers robust itch-evoked scratching, which induces AD skin phenotype (72). Therefore TSLP is thought to drive AD (73). Tezepelumab is a monoclonal antibody that targets TSLP. The data from double-blind, placebo-controlled phase 2a studies done by Simpson et al. showed that 64.7% of tezepelumab plus TCS-treated patients compared with 48.2% of placebo group achieved EASI-50 [(38); Table 1], followed by a greater improvement seen at week 16 (*post hoc*). EASI-75 and EASI-90 also show a similar trend (see Supplementary Table S1). Furthermore, numerical improvements were observed in SCORAD50 and SCORAD75 at week 12 (41.0% vs. 29.4% for SCORAD50, and 9.8% vs. 7.4% for SCORAD75), whereas the peak pruritus NRS scores were lower for patients treated with tezepelumab and TCS. However, there was no substantial difference in SCORAD at week 16. With reference to the TEAEs, 8.9% of patients with tezepelumab plus TCS versus 12.7% of patients with placebo plus TCS experienced a TEAE. The most frequent was nasopharyngitis, which was observedin 23.3% patients receiving tezepelumab plus TCS and 20.0% placebo plus TCS-treated patients, and the injection-site erythema was reported by 5.4% of drug-treated group but 0% in placebo group.

## Anti-IL-17 antibody drugs

Interleukin-17 (IL-17) is an essential proinflammatory cytokine, which is mainly secreted by the CD4+ helper T cells and is associated with the pathogenesis of inflammatory diseases, including atopic dermatitis. The IL-17 axis thereby is an important pathway for targeted therapy for AD (74). Secukinumab is a humanized anti-IL-17A monoclonal antibody. In 2020, Benjamin et al. reported a randomized, double-blind, placebo-controlled phase 2 study (39). Totally 41 patients were involved in this 16 weeks study, including 27 people received secukinumab and others received placebo. SCORAD and EASI scores were recorded in every 4 weeks, however, the improvement was not significant. While, the percentage improvement of SCORAD for drug-treated group and placebo group in week 16 were − 7.6 vs. -8.5, the percentage changes in EASI were − 6.1 and − 27.3, respectively. A *post hoc* analysis of Asian patients was also conducted by them as a higher TH17 activation was demonstrated in these patients, however, a similarly no significant changes were found. Adverse events occurred in this trial were orbital cellulitis, upper respiratory infection and streptococcal pharyngitis, both data were the same for secukinumab and placebo group (4% vs. 0%).

## Anti-IL-5 antibody drugs

Mepolizumab is a humanized immunoglobulin monoclonal antibody that binds to IL-5 (Figure 1). A multicenter, randomized, double-blind, placebo-controlled, parallel-group, phase 2 study was carried by Kang et al. (40). According to the result from this clinical trial, mean score for the placebo group demonstrated a modest improvement in EASI score (−30.5%) whereas the mepolizumab-treated group reflected a greater improvement (−43.9%) and this trend continued through Week 12 (−22.4% vs. −42.5%). Similar mean scores between treatment groups were observed at Week 16 (−32.3% vs. −31.9%), and while an increase in mean score was observed at Week 20 in the placebo group, there was a further decrease in the mepolizumab group (1.3% vs. −63.9%). For the endpoint of IGA, only 2 of 18 (11%) patients receiving mepolizumab and none of the 16 patients receiving placebo achieved 0/1 at week 16 (Table 1). In this study, no serious adverse event was reported.

## Anti-OX40 antibody drugs

Preclinical studies of skin inflammation and asthma models have supported that OX40-OX40L signaling interactions are pivotal to the efficiency of the responses that are regulated by memory Th2 cells [(75); Figure 1]. Therefore anti-OX40 antibodies are potential therapeutic treatments for moderate-to-severe AD patients, as they target the immunopathogenic pathways (76). A phase 1 study carried by Nakagawa et al. revealed that patients with rocatinlimab showed improvements in both EASI and IGA, see in Table 1 (41). The pruritus NRS score decreased over time from 6.8 ± 2.2 to 2.4 ± 2.3 at the end of the trial. Later in 2023, Guttman-Yassky et al. conducted a multicentre, double-blind, placebo-controlled phase 2b investigation to evaluate the efficacy and safety of rocatinlimab. From this study a significant least-squares mean percent reductions in EASI score at week 16 could be observed in all rocatinlimab groups (rocatinlimab 150 mg every 4 weeks −48.3; rocatinlimab 600 mg every 4 weeks −49·7; rocatinlimab 300 mg every 2 weeks −61.1; and rocatinlimab 600 mg every 2 weeks −57.4), compared with placebo (−15.0) (Table 1; Supplementary Table S1). Furthermore, improvements in disease severity measures and patient-reported outcomes largely remained during the off-treatment follow-up period. The adverse events occurred commonly were pyrexia, nasopharyngitis, chills, headache, aphthours ulcer and nausea (42).

In a phase 2a study in 2019, GBR830, which is another humanized mAb against OX40, was investigated. Patients were divided into 2 groups, 62 in ITT and 40 in BAS population, respectively. The results indicated that GBR830 was well tolerated, with a greater proportion of drug-treated patients achieving 50 and 75% reduction in EASI. A higher reduction in SCORAD score in day 71 was also observed in GBR830 group than in placebo group (−45.4 ± 26.9 vs. -31.0 ± 16.9 for ITT and − 44.0 ± 27.6 vs. -31.4 ± 18.2 for BAS). Moreover, the mean change from baseline in pruritus NRS score in week 10 showed the similar pattern, which is −2.7 ± 2.5 vs. -1.5 ± 1.6 and − 3.0 ± 2.6 vs. -1.3 ± 1.6 for each population. Lesional skin, epidermal hyperplasia and proliferation were reduced significantly after treating with GBR830. However, 63% of participants in GBR830 group reported TEAEs, which was numerically identical to that in placebo group, including headache, nasopharyngitis, upper respirator tract infection, fatigue, etc. (43).

Amlitelimab inhibits OX40-OX40L pathway by binding OX40L and blocking interaction with OX40. Weidinger et al. evaluated its tolerability and safety by conducting a phase 2a, randomized, placebo-controlled, double-blind multicentre trial. According to this investigation, while a higher proportion of clinically meaningful improvements in EASI were seen in amlitelimab low dose and high dose groups than placebo group (−80.12% and −69.97% vs. −49.37%), more patients in drug-treated group achieved vIGA 0/1 at week 16 (44% for low dose and 37% for high dose vs. 8%), and this clinical response sustained to week 36 (44). Overall, amlitelimab was thought to be well tolerated in this trial. Despite there was a death in the post-week 16, this was considered to be unrelated to amlitelimab. Thereby, with only one related serious adverse event reported during the study, amlitelimab had an remarkable safe index (44).

## Conclusion

To date, antibody drugs have made a great progress, with a growing number of patient, especially for those with moderate-to-severe atopic dermatitis, using them as their treatments. While the antibody drugs that target IL-4 and IL-13 showed a significant efficacy in treating AD, others such as tezepelumab, omalizumab and nemolizumab have been tested and shown to be effective. However, problems still exist, such as common adverse events, mere improvement in the outcomes and the high expenses. Therefore, researchers are now finding new targets or developing new antibody drugs to solve these defects. This article reviews the vast majority of antibody drugs and indicates their results from clinical trials, side effects and current stage, thus facilitating researchers to conduct in-depth comparative studies on drugs with the same targets, while providing basis for clinical appliances.

## Author contributions

MC contributed to conception and design of the study. GZ and YH wrote all versions of the manuscript, graph, and tables. All authors contributed to manuscript revision, read, and approved the submitted version.

## Funding

Peking University Medicine Seed Fund for Interdisciplinary Research supported by “the Fundamental Research Funds for the Central Universities” (Nos. BMU2021MX021 and BMU2022MX017). This work was supported by the National Natural Science Foundation of China (81603119) and the Natural Science Foundation of Beijing Municipality (7174316).

## Conflict of interest

The authors declare that the research was conducted in the absence of any commercial or financial relationships that could be construed as a potential conflict of interest.

## Publisher’s note

All claims expressed in this article are solely those of the authors and do not necessarily represent those of their affiliated organizations, or those of the publisher, the editors and the reviewers. Any product that may be evaluated in this article, or claim that may be made by its manufacturer, is not guaranteed or endorsed by the publisher.
